# Associations Between Vascular Endothelial Growth Factor, Major Depressive Episode and Response to Electroconvulsive Therapy: A Meta-Analysis

**DOI:** 10.1192/j.eurpsy.2025.349

**Published:** 2025-08-26

**Authors:** I. Borja De Oliveira, G. A. M. Alves, D. Soler Lopes, A. V. de Vasconcelos, A. L. Stephany, F. Reis Soares, M. Kraide Piedade de Abreu, M. C. Carbajal Tamez, J. Quevedo, M. Teranishi

**Affiliations:** 1 School of Medicine, University of São Paulo, São Paulo, Brazil; 2 Humanitas University, Milan, Italy; 3 Lancaster University, Lancaster, United Kingdom; 4 Afya College of Medical Sciences of Santa Inês, Santa Inês; 5 São Paulo Association for the Development of Medicine, São Paulo; 6 Pontifical Catholic University of Goias, Goiania, Brazil; 7 Department of Psychiatry and Behavioral Sciences at McGovern Medical School; 8 Center for Interventional Psychiatry, Faillace Department of Psychiatry and Behavioral Sciences, McGovern Medical School, The University of Texas Health Science Center at Houston, Houston, United States; 9 Università degli Studi di Milano, Milan, Italy

## Abstract

**Introduction:**

Major depressive episodes (MDEs) occur in mood disorders such as major depressive disorder (MDD) and bipolar disorder (BD), affecting nearly one in four U.S. adults over their lifetime. The neurotrophic hypothesis suggests that disruptions in growth factor signaling contribute to MDEs. While brain-derived neurotrophic factor (BDNF) is well-studied, vascular endothelial growth factor (VEGF) may also play a crucial role, though evidence of its association with MDEs is inconsistent. Understanding VEGF is important for identifying predictors of treatment outcomes, such as those related to electroconvulsive therapy (ECT). This study explores the relationship between VEGF and MDEs, focusing on implications for ECT effects.

**Objectives:**

To consolidate evidence from studies evaluating the association between VEGF and ECT outcomes in patients experiencing an MDE.

**Methods:**

A systematic search for published clinical trials and cohort studies was conducted on August 13, 2024, using keywords including ECT, VEGF, MDE, and mood disorders, with no language or publication date restrictions. We selected studies enrolling patients in a current MDE related to MDD or BD, excluding those focused on manic episodes. A fixed-effects or random-effects model was applied. Subgroup analyses were performed to investigate the data further.

**Results:**

Seven studies involving 621 participants (61.9% female; mean age: 50.2 years) were preselected. Six studies measured plasma VEGF levels; one assessed cerebrospinal fluid (CSF) levels. Plasma VEGF levels did not differ significantly between healthy controls and MDE patients, either before (SMD = 0.02 [−0.17; 0.21], p = 0.84, I² = 0%) or after ECT (SMD = 0.11 [−0.21; 0.44], p = 0.50, I² = 0%). Of the five studies reporting post-ECT VEGF levels, three found a significant increase from baseline. A significant correlation was observed between baseline plasma VEGF levels and depression response to ECT (r = 0.34, Z = 4.92, p < 0.0001, I² = 0%). Of the five studies examining increased VEGF levels after ECT and symptom reduction, only one found a significant association. A sensitivity analysis indicated substantial heterogeneity when including the CSF study.

**Conclusions:**

Plasma VEGF levels were not significantly different in MDE patients compared to healthy controls, either before or after ECT. Baseline plasma VEGF levels positively correlated with ECT treatment response, suggesting they may provide neurotrophic support and predict outcomes. Despite robust findings and minimal heterogeneity, this analysis was limited by the low number of studies and small sample sizes. Further research is needed to explore the association between MDEs and VEGF, especially in the CSF, and to clarify the role of baseline VEGF in ECT treatment response.

**Disclosure of Interest:**

I. Borja De Oliveira: None Declared, G. A. M. Alves: None Declared, D. Soler Lopes: None Declared, A. de Vasconcelos: None Declared, A. Stephany: None Declared, F. Reis Soares: None Declared, M. Kraide Piedade de Abreu: None Declared, M. Carbajal Tamez: None Declared, J. Quevedo Shareolder of: Instituto de Neurociencias Dr. Joao Quevedo, Grant / Research support from: LivaNova; and receives copyrights from Artmed Editora, Artmed Panamericana, and Elsevier/Academic Press, Consultant of: EMS, Libbs, and Eurofarma, Speakers bureau of: Myriad Neuroscience and AbbVie., M. Teranishi: None Declared

